# Diagnostic accuracy of cardiovascular magnetic resonance for patients with suspected cardiac amyloidosis: a systematic review and meta-analysis

**DOI:** 10.1186/s12872-016-0311-6

**Published:** 2016-06-07

**Authors:** Lei Zhao, Zhuang Tian, Quan Fang

**Affiliations:** Department of Cardiology, Peking Union Medical College Hospital, Peking Union Medical College and Chinese Academy of Medical Sciences, No.1 Shuaifuyuan, Wangfujing, Dongcheng District Beijing, 100730 China

**Keywords:** Cardiac amyloidosis, Cardiac magnetic resonance, LGE, Meta-analysis

## Abstract

**Background:**

This study is a systematic review and meta-analysis of the diagnostic value of cardiovascular magnetic resonance (CMR) in cardiac amyloidosis (CA).

**Methods:**

A wide variety of electronic databases were searched for studies of CMR that reported the diagnostic accuracy in patients with suspected CA. Research manuscripts were subjected to further systematic review and meta-analysis. Methodological evaluation was performed under the guidance of the Quality Assessment of Diagnostic Accuracy Studies −2 (QUADAS–2). Heterogeneity was assessed, and a random-effects model was used to assess the diagnostic effects of CMR on pooled sensitivity, pooled specificity, and summary receiver operating characteristics (SROC).

**Results:**

Seven studies that reported the performance of CMR for CA were included in the present systematic review, among which five studies (257 patients) that evaluated the diagnostic accuracy of late gadolinium enhancement (LGE) CMR were analyzed in the present meta-analysis. Heterogeneity was observed only in specificity. A summary sensitivity and specificity of 85 % (95 % CI: 77–91 %) and 92 % (95 % CI: 83–97 %) indicated a high diagnostic accuracy of LGE for CA. The AUC of SROC curve was 0.9530, suggesting that LGE is an effective way of diagnosing patients with possible cardiac involvement in amyloidosis.

**Conclusions:**

LGE–CMR seems to have a relatively high diagnostic accuracy for amyloidosis patients with possible cardiac involvement. Combined CMR techniques may provide important information for the selection of suitable therapy.

**Electronic supplementary material:**

The online version of this article (doi:10.1186/s12872-016-0311-6) contains supplementary material, which is available to authorized users.

## Background

The heart, like any organ in the body is susceptible to amyloid deposition. When this occurs in the heart, cardiac amyloidosis (CA) results. Although more than 30 types of protein can cause amyloid, only two types commonly deposit in the ventricular myocardium: amyloid light chain (AL) and amyloid transthyretin (ATTR). The heart is sometimes the only or early manifestation of systemic amyloidosis [1]. Deposition of amyloid precursor proteins which then aggregate into amyloid fibrils in the extracellular space causes separation and distortion of the existing tissues and eventually causes irreversible cardiac dysfunction [2, 3]. This may occur in the myocardium, pericardium, small vessels and conduction system. The result is a restrictive cardiomyopathy with early diastolic dysfunction and later systolic dysfunction, conduction disease including sudden death and, occasionally ischaemia (with arterial involvement) [4].

CA is relatively under-diagnosed, especially wild type ATTR in older people with multisystem diseases. One potential reason is that the symptoms and signs of CA such as fatigue, low exercise tolerance and occasionally syncope with progressive diastolic and systolic dysfunction may be nonspecific, particularly in the early phases, resulting in delayed diagnosis and potentially missed therapeutic options. New treatments are available targeting the precursor proteins, including newer chemotherapy regimes and a variety of approaches to reduce transthyretin as a precursor, with further developments in the preclinical phase [5, 6]. For this reason, earlier diagnosis of CA is a major clinical goal [7].

Currently, the main steps in the clinical evaluation of patients with CA involve suspicion, detection, and classification. Suspicion may be raised by multisystem involvement (carpal tunnel, renal impairment etc.). Laboratory investigations may confirm the diagnosis, classify the CA, trace the specific protein precursor and appraise the stage of cardiac involvement including pump function [8]. Currently, the most common examination procedures used to evaluate patients or conditions associated with CA include endomyocardial biopsy (EMB), electrocardiography (ECG), echocardiography and cardiac magnetic resonance (CMR) with some nuclear tests contributing (bone scintigraphy for ATTR, SAP scan for AL - but these are not widely available).

In CA, myocardial tissue histology stands as the gold standard, revealing the accumulation of amyloid proteins and fibers in the extracellular space. Histologically, EMB is initially stained using Congo Red where deposits may look pale red but show apple-green birefringence under cross-polarized light. Immunohistochemistry (IHC) or mass-spectrocopy can definitively subtype the amyloid fibrils [4]. However, the invasive nature of EMB and related high-risk complications are barriers, as is the specialist nature of IHC and mass spectroscopy. Besides, as the lack of standardization on the definition of what constitutes CA, in some cases these is a need to use some ancillary imaging and clinical findings to ensure the pathological diagnosis. In addition, EMB is classified in the class IIa recommendation [9].

ECG Low -voltages is a classical feature of CA [10]. But it provides a low -sensitivity and -specificity diagnosis of CA. This may be due in part to interference from other coexisting conditions, such as obesity, hypothyroidism, and pulmonary diseases, but up to 25 % of ATTR subjects have left ventricular hypertrophy. Under echocardiograph, the CA classical feature (a "brilliant" speckled appearance of myocardium) is a poor discriminator. Biventricular hypertrophy with disproportionate long axis impairment and restriction is more specific, but still has overlap, particularly in early disease [11].

CMR, a noninvasive mean of assessing amyloid burden, makes use of a superconducting magnet with ECG gating to measure the structure and function of the heart analogous to echocardiography. In addition, myocardial tissue characterisation detects tissue pathology [12]. In particular, the late gadolinium enhancement (LGE) has a characteristic pattern in amyloid with subendocardial, later transmural LGE and difficulty nulling. This latter issue appears resolved with the phase-sensitive inversion recovery (PSIR) LGE sequence and it has potential to be a key technique for the diagnosis of patients with potential CA [13]. Several studies have compared the diagnostic accuracy of CMR to that of EMB. However, they focused on different parameters and used different cutoff values in diagnostic tests, which limit their usefulness in the assessment of CA. For this reason, a systematic review was conducted to help evaluate the diagnostic aspects of CMR in patients with suspected CA.

## Methods

### Data sources

This systematic review was carried out under the guideline of the Cochrane Collaboration’s Diagnostic Test Accuracy Group. Search strategies were developed to identify all the relevant studies published in the Pubmed, Embase, the Cochrane Library, Biosis Preview, ISI Web of Science, and China National Knowledge Infrastructure up to April 20, 2015. Medical subject headings and full text were searched for references to CA and magnetic resonance imaging to systematically locate these publications in any language. The details of the search strategies are provided in the additional files. The citations of eligible publications and reviews were also checked. Requirements were limited to human trials.

### Study selection and data collection

The first author (LZ) conducted the initial literature search of titles and abstracts. Upon further scrutiny, two reviewers (LZ, ZT) led the retrieval of the full texts of relevant manuscripts independently. A third reviewer (QF) resolved all the disagreements and discrepancies that occurred during these processes through deep discussions. The following inclusion criteria were defined in advance: (1) a diagnostic accuracy test was included; (2) patients with at least suspected CA; (3) the diagnostic accuracy of CMR was evaluated regardless of acquisition protocol used; (4) EMB, clinical criteria, or both served as the reference tests; (5) the absolute numbers of true positive, false positive, true negative, and false negative results could be derived. All the articles that met these criteria were considered eligible. Duplicate and overlapping publications were excluded. Conference abstracts and research manuscripts that could not be obtained in their entirety or from which essential information was missing were also excluded. Diagnostic accuracy details, study methods, results, and additional information on patients and procedures were extracted from each primary study independently by two reviewers (LZ, ZT).

### Assessment of risk of bias

Two reviewers (LZ, ZT) appraised the methodological quality based on the form suggested by the Quality Assessment of Diagnostic Accuracy Studies–2 (QUADAS–2), which has main parts: patient selection, index test, reference standards, and the flow and timing [14]. Each part involves the assessment of risk of bias (low, high, or unclear) and first 3 parts also need to evaluate the clinical applicability. In cases of disagreement, 2 reviewers would reach a consensus after a thorough discussion with a third person (QF).

### Statistical analysis

Statistical analyses were conducted using MetaDisc 1.4 software and Review Manager software version 5.3. First, heterogeneity in included manuscripts was assessed in terms of Cochran Q chi-square tests and I^2^ statistics. In cases of significant heterogeneity, the random-effects model was suitable to pool several parameters and sensitivity analysis was used to investigate potential sources targeting risk bias calculated by QUADAS–2. In other cases, the Mantel-Haenszel method was used to establish a fixed-effects model during the evaluation process. In cases of implicit threshold effects, the Spearman rank correlation was taken into consideration. Subgroup analysis determined if there was a correlation between sensitivity and specificity. Second, positive likelihood ratios (PLR), negative likelihood ratios (NLR), and diagnostic odds ratio (DOR), and their 95 % confidence intervals (CI) were estimated based on pooled sensitivity and specificity. In order to estimate the overall diagnostic effect, a summary receiver operating characteristic (SROC) curve was established, with pertinent areas. Whenever a study’s data contained zero counts, 0.5 was added as a continuity correction. Any significantly statistical calculating was at a two-sided 0.05 level.

## Results

**Fig. 1:**
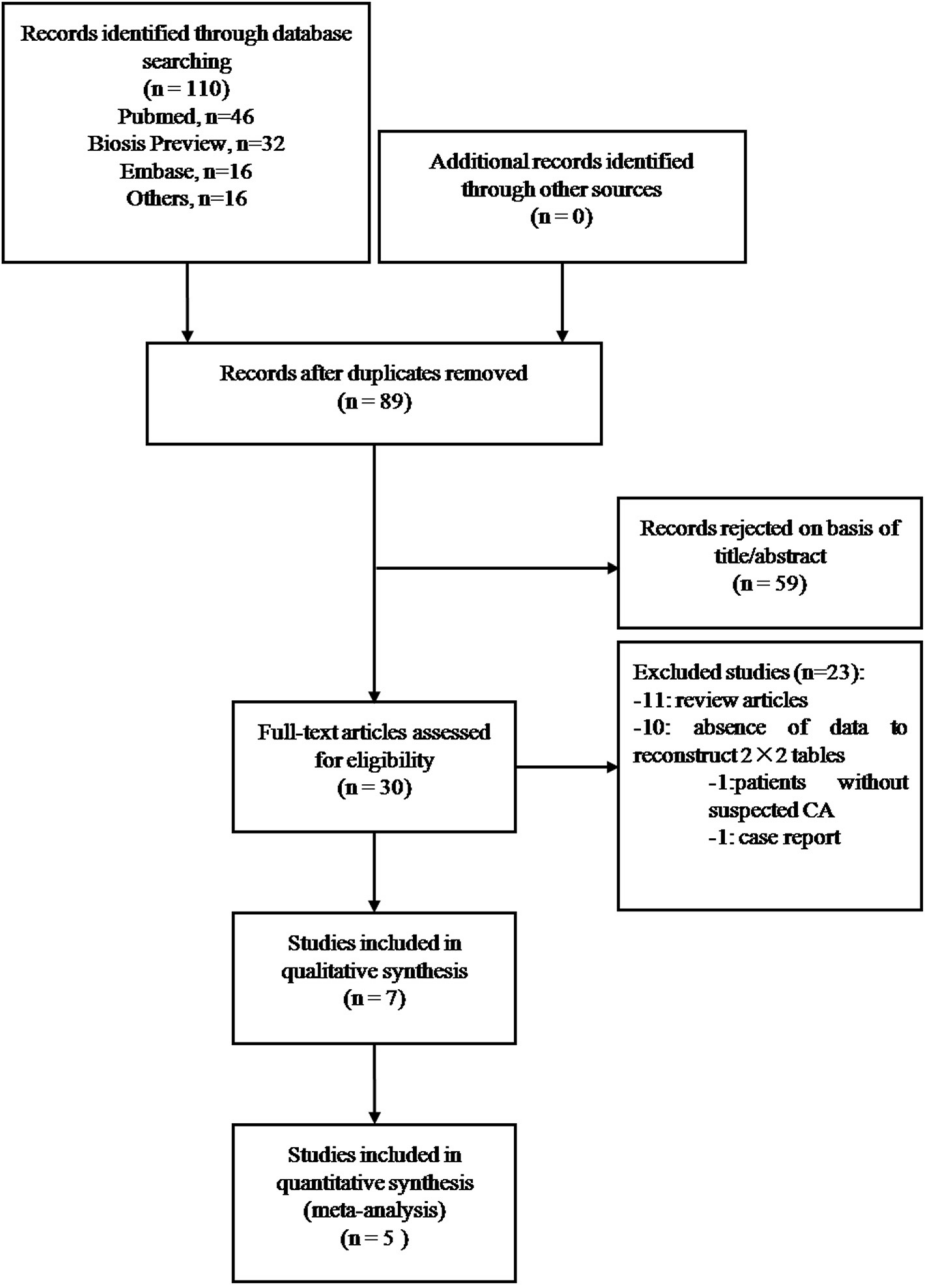
Flow chart of selection process

The systematical electronic search identified 110 publications, of which 46, 32, 16, 10, 5, and 1 were found in Pubmed, Biosis Preview, Embase, ISI Web of Science, Cochrane Library and China National Knowledge Infrastructure. Figure 1 outlines the process of manuscripts screening. Here, 21 studies were excluded because of duplicate records. After the removal of 59 studies whose titles or abstracts did not meet the inclusion criteria, only 30 full-text articles were retained for further evaluation. Of the 30 potentially appropriate publications, only 7 were included in the systematic review [15–21]. Most of the excluded studies were reviews or did not provide absolute figures. The meta-analysis was performed on 5 studies that focused on the LGE performance [17–21].

### Methodological evaluation

**Fig. 2:**
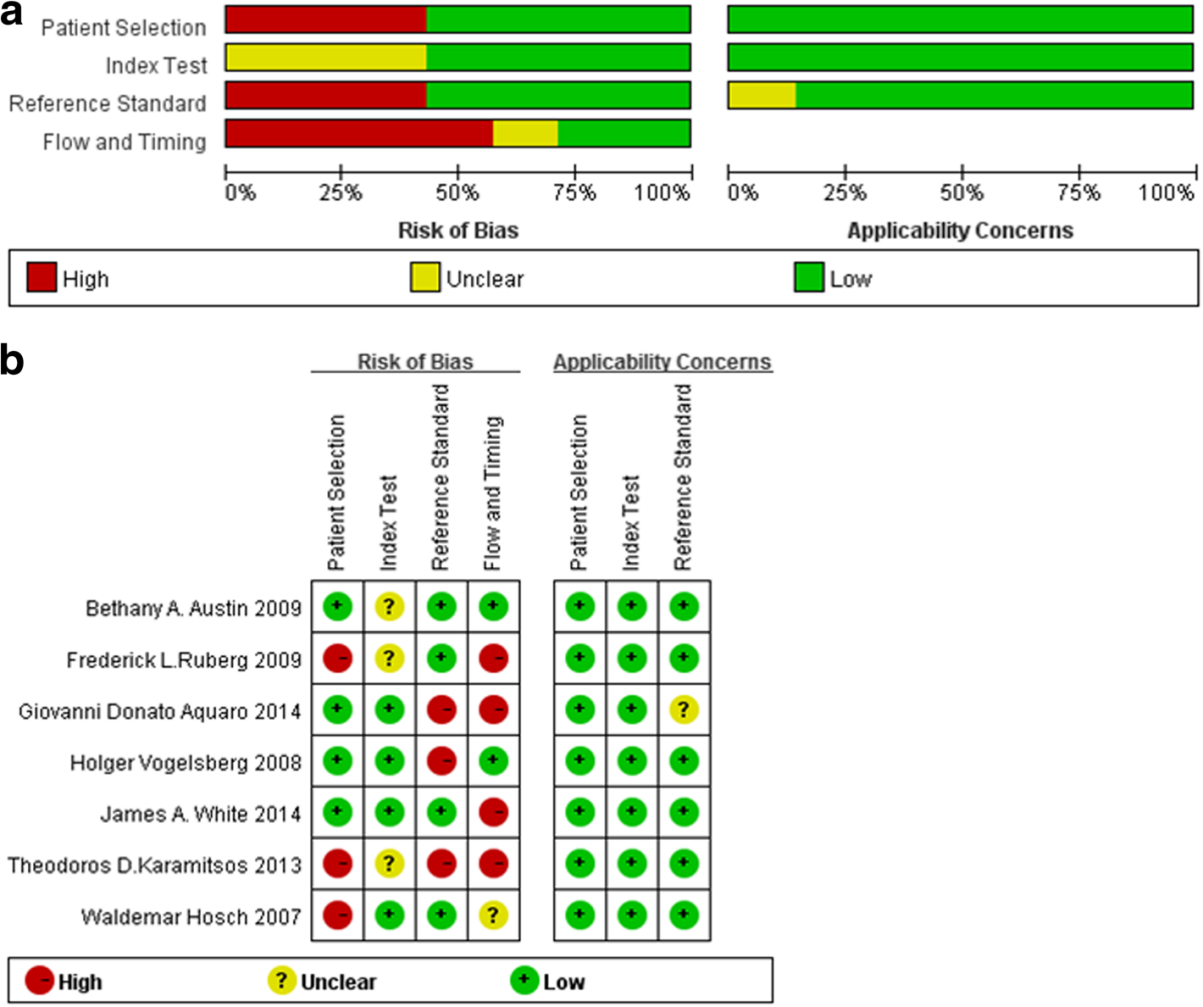
Assessment of methodological quality according to QUADAS-2

Figure 2 shows the graphs of risk bias and concerns about clinical application evaluated by QUADAS–2. In the patient selection domain, one report did not state that patients were enrolled in a consecutive pattern [20]. Another study lacked relevant information on patient sampling [16]. The Karamitsos and Hosch studies both used a case–control design [15]. In terms of an inappropriate exclusion, both Karamitsos and Hosch’s studies were unclear. Regarding interpretation of the CMR results, 2 studies did not clearly state whether they were conducted before knowing the results of EMB, the one by Karamitsos and another one by Austin [19]. LGE–CMR was used in 6 studies, and one study used MR-relaxometry to evaluate T1 and T2 relaxation time (RT), which established a cut-off value of 1273 ms of T1-RT to diagnose CA with a high sensitivity and specificity [15]. The other 6 studies detailed the LGE-CMR procedures and defined amyloid LGE pattern using long and short axes images in the ventricular area without coronary artery distribution [16–21]. No concerns about the included patients going against the inclusion criteria.

There were 2 studies that did not clearly report that the EMB evaluation was blinded to CMR results [19, 21]. Another study stated it was done without blindness [17]. In terms of the reference tests, some patients could not be classified clearly using clinical criteria alone [16, 21]. This kind of study involved high risk bias and uncertainty regarding use in the reference domain.

One study was discarded because of incomplete data for 2 × 2 table [16]. The interval between CMR and the reference test were only clearly stated in 3 studies [17, 19, 21]. But these 3 studies together did not use the same reference test on all the patients. Some patients were evaluated by EMB and others were clinically assessed using ECG, echocardiography, or both. In the study by White, only 25 (28 %) patients with suspected CA were included in the final diagnostic evaluation [18].

Overall, all the included studies involved little concerns regarding applicability and remaining 5 studies were here considered methodologically correct regardless of risk bias [17–21].

### Study characteristics

The baseline characteristics of these included studies are shown in Table 1 [15–21]. A total of 485 subjects were recruited in the final systematic analysis. These manuscripts were published during the period from 2007 to 2014 and four of them were prospective studies [15, 18, 20, 21]. Patients involved in individual studies are summarized concisely in Table 1. The sample size ranged from 28 to 106 and the mean age was similarly around 62 years old. Mean time intervals were only available in three studies [17, 19, 21]. Six studies used EMB as a reference test [15, 17–21]. Another one used echocardiography and clinical features as to classify patients [16]. All studies used 1.5-T scanners to perform the CMR, but only five of them evaluated the diagnostic accuracy of LGE by calculating sensitivity and specificity [17–21]. No adverse events were reported in the examinations.

**Table 1:** Characteristics of included studies

Study	Year	Country	Study design	Population	Age, yrs	Male, n	Mean time interval^a^	Blind^b^	Reference test	Index measured using CMR	Field strength
Hosch et al.	2007	Germany	PC	19 with newly diagnosed CA and 9 patients with systemic amyloidosis but without CA and 10 healthy, age-matched control	59 ± 6.1	14	NA	Y	EMB	T1 and T2 RT	1.5 T
Vogelsberg et al.	2008	Germany	NA	33 subjects with suspected CA	64 ± 13	NA	Same day	Y	EMB	Amyloid LGE pattern	1.5 T
Austin et al.	2009	USA	RC	47 subjects with suspected CA	62	33	24–48 h	N	EMB	LGE; DHE	1.5 T
Ruberg et al.	2009	USA	PC	28 patients with systemic AL amyloidosis	62 ± 11	20	NA	Y	EMB or clinical criteria	LGE	1.5 T
Karamitsos et al.	2013	UK/Canada	NA	53 patients with systemic (primary) AL amyloidosis and 17 patients with aortic stenosis and 36 normal volunteers	63 ± 10^c^	69	NA	Y	Echocardiography and clinical features	Noncontrast T1 mapping; LGE	1.5 T
Aquaro et al.	2014	Italy	PC	59 patients with a previous diagnosis of systemic AL amyloidosis and 20 healthy control subjects	69 ± 10	36	Same day	Y	Echocardiographic criteria and/or ECG criteria or EMB	SID; conventional LGE	1.5 T
White et al.	2014	USA	PC	90 patients with suspected CA and 64 hypertensive patients with LVH	62 ± 13	52	NA	Y	EMB	LGE pattern: Global HE	1.5 T

*AL* amyloid light-chain, *CA* cardiac amyloidosis, *DHE* delayed hyper-enhancement, *ECG* echocardiography, *HE* hyperenhancement, *EMB* endomyocardial biopsy, *LGE* late gadolinium enhancement, *LVH* left ventricular hypertrophy, *N* no, *NA* not available, *PC* prospective cohort, *RC* retrospective cohort, *RT* relaxation times, *SID* signal intensity decay

^a^Time interval between reference tests and cardiac magnetic resonance

^b^The CMR results were interpreted blind to the results of the reference tests or the reference tests were performed without knowing the results of cardiac magnetic resonance

^c^Amyloid patients with definite cardiac involvement

### Diagnostic performance of CMR for CA

Five studies contained absolute figures for 2 × 2 tables with respect to LGE and were included in the meta-analysis (Table 2) [17–21]. Individual studies are shown on forest plots focusing on sensitivity and specificity. The *χ*^2^ test for sensitivity and specificity produced a *P* value of 0.2003 and 0.0581 with I^2^ values of 33.2 % and 56.1 %, respectively. In cases with a detectable threshold effect, the Spearman rank correlation between the sensitivity and 1-specifity was performed and the correlation coefficient was 0.8 with a *P* value 0.104 (Additional files 1 and 2). Because the inconsistency index I^2^ was used to quantify the heterogeneity, an I^2^ value of 56.1 % was considered moderate (50–74 %). For this reason, the random-effects model was used for the meta-analysis.

**Table 2:** Cardiac magnetic resonance evaluation of the included studies in the systematic review

Study	Number of subjects	Index measured using CMR	TF	FP	FN	TN	Sensitivity (%)	Specificity (%)	PPV (%)	NPV (%)	AUC
Hosch et al.	38	T1-RT	16^a^	0^a^	3^a^	10^a^	84^a^	100^a^	NA^a^	NA^a^	0.96^a^
			16^b^	1^b^	3^b^	8^b^	84^b^	89^b^	NA^b^	NA^b^	0.89^b^
Vogelsberg et al.	33	LGE	12	1	3	17	80	94	92	85	NA
Austin et al.	47	LGE	15	2	2	19	88	90	88	90	NA
Ruberg et al.	28	LGE	18	1	3	6	86	86	95	67	NA
Karamitsos et al.	106	Noncontrast T1 mapping	36	6	3	61	92	91	NA	NA	NA
Aquaro et al.	79	SID	51	2	1	26	98	93	96	96	NA
		LGE	42	0	10	27	81	100	100	73	NA
White et al.	154	LGE	15	3	0	7	93	70	NA	NA	NA

*AUC* area under receiver-operating characteristic curve, *FN* false negative, *FP* false positive, *TN* true negative, *TP* true positive, *PPV* positive predictive value, *NA* not available, *NPV* negative predictive value, *LGE* late gadolinium enhancement, *RT* relaxation time, *SID* signal intensity decay

^a^CA patients compared with the healthy, age-matched control group

^b^CA patients compared with the patient group with systemic amyloidosis but without cardiac involvement

**Fig. 3:**
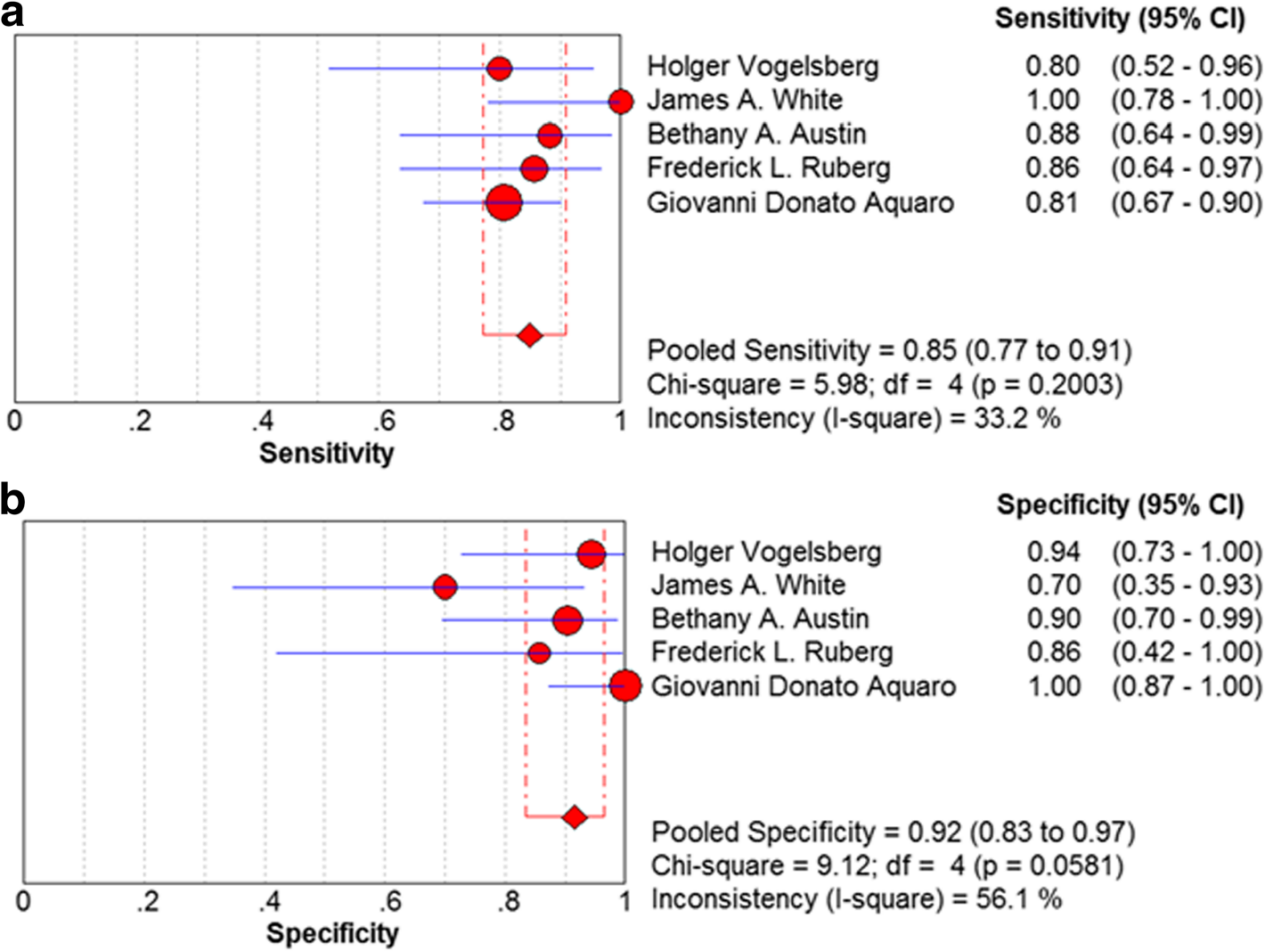
Forest plot evaluating the sensitivity and specificity of diagnostic performance of late gadolinium enhancement in included studies. CI, confidence interval

**Fig. 4:**
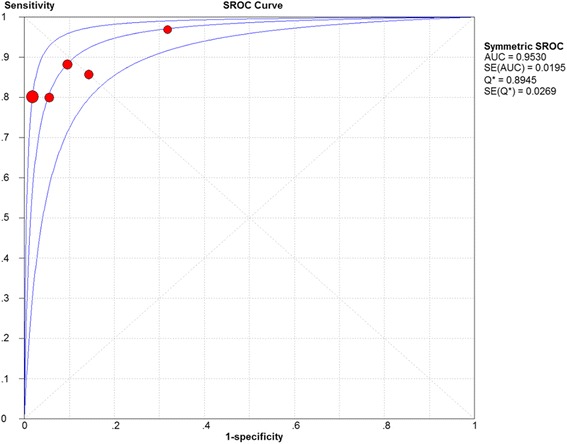
Summary receiver operator characteristics (SROC) of late gadolinium enhancement on summary estimates of sensitivity and specificity. AUC, area under the curve; SE, standard error

**Fig. 5:**
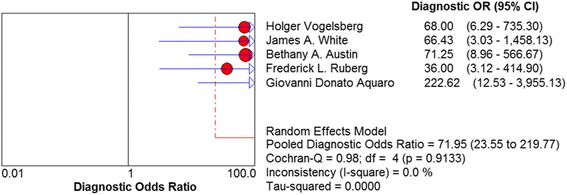
Forest plot presenting the pooled diagnostic odds ratio of late gadolinium enhancement. CI, confidence interval

As shown in Fig. 3, the pooled sensitivity and specificity of LGE-CMR were 85 % (95 % CI: 77–93 %) and 92 % (95 % CI: 83–97 %). The pooled positive likelihood ratio was 7.481 (95 % CI: 2.835–19.739) and the negative one was 0.183 (95% CI: 0.121–0.277). Figure 4 shows the SROC curve for LEG-CMR, suggesting the LGE is an effective mean of diagnosing patients with amyloidosis and potential cardiac involvement. These results also corresponded to the pooled diagnostic odd ratio (Fig. 5). There was some residual heterogeneity in the diagnostic accuracy. Sensitivity analysis was conducted to determine the source of heterogeneity. After removing the study by Aquaro which involved high risk bias in the methodological evaluation, results showed the specificity have an I^2^ value of 9.3 % and *P* value of 0.3467 [21]. However, this did not change the direction or magnitude of the pooled estimates, which means that the results of relatively good quality. Because only 5 publications were included, no funnel plot was constructed to assess publication bias.

## Discussion

Amyloidosis, when it affects the heart, has a poor prognosis. Better diagnostic tests would permit earlier detection and therefore targeted intervention to improve outcomes. Many modalities have been used in clinical settings to detect and evaluate CA [22]. The most common methods are ECG and echocardiography. These tools are convenient and available in most medical facilities in which the EMB is not in common use. However, both methods detect CA with a relatively low accuracy. ECG evaluation is not suitable for patients with pulmonary disorders but many patients with cardiomyopathy, especially elderly patients, are likely to be involved in cardiopulmonary diseases. Echocardiography can detect the advanced cases [23]. However, it cannot easily assess earlier cases or differentiate CA from hypertrophic cardiomyopathy because the characteristics in echocardiography are thick-walled ventricles with speckled myocardia [4, 23]. EMB is considered the gold standard in the diagnosis of CA. However, the unavailability, relatively high risks and clinical complications may hinder its widespread use in clinical settings. CMR provides a third option in the evaluation of the heart’s structure and assessment of its diastolic function. It is more precise and more accurate, which are regarded as the hallmarks of good non-invasive methods capable of assessing myocardial scars [13, 24].

The key advantage of using CMR to detect the CA is its unique ability to describe the characteristics of heart, which can be done particularly using LGE [25]. After taking the gadolinium-based contrast, patients with CA presented typical global subendocardial enhancement patterns, which are closely associated with interstitial amyloid burden and often used to qualitatively detect focal fibrosis [26]. This meta-analysis of LGE–CMR showed that the diagnostic accuracy increased with a summary sensitivity of 85 % and a summary specificity of 92 %. In terms of the heart tissue characterization, LGE can distinguish CA patients from healthy controls with a summary positive likelihood ratio of 7.481 (95 % CI: 2.835–19.739). The summary diagnostic odds ratio of 71.945 detected here (95 % CI: 23.552–219.77) indicated that expressed the test was more accurate in the differential diagnosis of CMR than other types of diagnosis. In this way, the values of LR+ and DOR favor of LEG–CMR in patients with suspected CA before EMB. Heterogeneity was observed across studies in terms of specificity, but not sensitivity. This could be partially due to the methodological quality of involved publications on reference tests choosing and flow and timing.

AL and ATTR are two types of amyloidosis typically associated with the heart. ATTR is often underdiagnosed and can be fatal. It also has particular variants in specific ethnic populations, which makes diagnosing and typing both necessary and challenging. Recently, several studies have reported that the new CMR techniques have better diagnostic accuracy in demonstrating the range of structural and functional changes in different types of CA [27]. T1 mapping, a new technique measuring myocardial intrinsic signal, was found to diagnose AL and ATTR very accurately [28, 29]. Native T1 levels were higher in ATTR patients than in controls and the area under the AUC for ATTR and AL patients with possible or definite cardiac involvement was 0.85 (95 % CI: 0.79–0.92) [29]. One study reviewed here evaluated the noncontrast myocardial T1, and the cut-off of 1020 ms yielded 92 % accuracy for identifying CA [16]. T1–RT reported by Hosch also diagnosed CA with a high sensitivity (84 %) and specificity (>89 %) with a cut-off value of ≥1273 ms [15]. The measurement of extracellular volume (ECV) measurement is popular in some centers [30, 31]. ECV is elevated in both AL and ATTR, which helps the evaluation of amyloid burden. It is also elevated in patients where routine examinations, including LGE, indicated no heart involvement. In the present systematic review, one study analyzed the myocardial signal intensity decay (SID) at the subendocardial level after gadolinium injection, showing a greater accuracy (96 %) and sensitivity (98 %) for CA assessment than LGE with a 269 HB threshold [21]. T1 values were higher in AL amyloid than in ATTR, but ECV was higher in ATTR [12]. This suggests that combined T1 and ECV techniques may attach more diagnostic accuracy to patients with cardiac involvement, quantify the amyloid burden to evaluate the pathologic conditions, and distinguish types of CA to help clinicians select the most appropriate treatment for each patient. When LGE combine with these advanced techniques, CMR could be a main technique for the diagnosis of patients with potential CA.

The current review has some limitations, which should be taken into consideration. The main one is the small number of studies included, which restricted deep exploration of potential sources of heterogeneity. The second limitation is that the typical amyloid LGE pattern varies across different series. Localized enhancement, diffuse transmural or patchy LGE enhancement was reported in different studies. In this review, only three studies described the diffuse, global subendocardial enhancement pattern as the amyloid pattern. The third limitation is that not all of the studies used the same reference tests. Not all patients underwent the same reference tests, introducing selection bias in the final results. Despite these limitations, the current work can present the current state of diagnostic tests of CMR for CA, which may help medical workers choose diagnostic methods efficiently and effectively.

## Conclusion

The current work indicates that the LGE is more accurate in the diagnosis of CA, which makes it valuable in the detection of amyloid deposit. However, when considering clinical cost-effectiveness and patients with claustrophobia, implanted cardiac devices and renal failure, CMR application value in CA needs further investigation.

## Abbreviations

AL, Amyloid light chain; ATTR, Amyloid transthyretin; AUC, Area under the curve; CA, Cardiac amyloidosis; CI, Confidence intervals; CMR, Cardiovascular magnetic resonance; DOR, Diagnostic odds ratio; ECG, Electrocardiography; ECV, Extracellular volume; EMB, Endomyocardial biopsy; LGE, Late gadolinium enhancement; NLR, Negative likelihood ratios; PLR, Positive likelihood ratios; PSIR, Phase-sensitive inversion recovery; QUADAS–2, Quality Assessment of Diagnostic Accuracy Studies–2; RT, Relaxation time; SID, Signal intensity decay; SROC, Summary receiver operating characteristics.

## Additional files

Additional file 1: Search strategy. (DOCX 142 kb) Additional file 2: Summary indexes in the meta-analysis. (DOCX 15 kb)
