# Supplementary material for: Diagnostic accuracy of cardiovascular magnetic resonance for patients with suspected cardiac amyloidosis: a systematic review and meta-analysis
Source: BMC Cardiovasc Disord. 2016 Jun 7;16:129. doi: 10.1186/s12872-016-0311-6 (PMC4897958; doi:10.1186/s12872-016-0311-6)
Supplement: Additional file 1: — Search strategy. (DOCX 142 kb) [file 12872_2016_311_MOESM1_ESM.docx]

**Additional file 1**

Search strategy

1. Pubmed

| Search | Query | Items found |
| --- | --- | --- |
| #7 | Search ((((((((heart diseases[MeSH Terms]) OR cardiomyopathy[MeSH Terms]) OR amyloid*[Title/Abstract]) OR CA[Title/Abstract])) AND ((((((((((Magnetic Resonance Imaging[Title/Abstract]) OR MRI[Title/Abstract]) OR "cardiovascular magnetic resonance"[Title/Abstract]) OR CMR[Title/Abstract]) OR LGE[Title/Abstract]) OR "late gadolinium enhancement"[Title/Abstract]) OR gadolinium[Text Word]) OR "delayed enhancement"[Title/Abstract]) OR "T1 time"[Title/Abstract]) OR "T1 mapping"[Title/Abstract])) AND (((diagnos*[Title/Abstract]) OR sensitivity[Title/Abstract]) OR specificity[Title/Abstract]))) AND cardiac amyloid*[Title/Abstract] Filters: Humans | 46 |
| #6 | Search ((((((((heart diseases[MeSH Terms]) OR cardiomyopathy[MeSH Terms]) OR amyloid*[Title/Abstract]) OR CA[Title/Abstract])) AND ((((((((((Magnetic Resonance Imaging[Title/Abstract]) OR MRI[Title/Abstract]) OR "cardiovascular magnetic resonance"[Title/Abstract]) OR CMR[Title/Abstract]) OR LGE[Title/Abstract]) OR "late gadolinium enhancement"[Title/Abstract]) OR gadolinium[Text Word]) OR "delayed enhancement"[Title/Abstract]) OR "T1 time"[Title/Abstract]) OR "T1 mapping"[Title/Abstract])) AND (((diagnos*[Title/Abstract]) OR sensitivity[Title/Abstract]) OR specificity[Title/Abstract]))) AND cardiac amyloid*[Title/Abstract] | 67 |
| #5 | Search cardiac amyloid*[Title/Abstract] | 1104 |
| #4 | Search ((((((heart diseases[MeSH Terms]) OR cardiomyopathy[MeSH Terms]) OR amyloid*[Title/Abstract]) OR CA[Title/Abstract])) AND ((((((((((Magnetic Resonance Imaging[Title/Abstract]) OR MRI[Title/Abstract]) OR "cardiovascular magnetic resonance"[Title/Abstract]) OR CMR[Title/Abstract]) OR LGE[Title/Abstract]) OR "late gadolinium enhancement"[Title/Abstract]) OR gadolinium[Text Word]) OR "delayed enhancement"[Title/Abstract]) OR "T1 time"[Title/Abstract]) OR "T1 mapping"[Title/Abstract])) AND (((diagnos*[Title/Abstract]) OR sensitivity[Title/Abstract]) OR specificity[Title/Abstract]) | 5927 |
| #3 | Search ((diagnos*[Title/Abstract]) OR sensitivity[Title/Abstract]) OR specificity[Title/Abstract] | 2395675 |
| #2 | Search (((((((((Magnetic Resonance Imaging[Title/Abstract]) OR MRI[Title/Abstract]) OR "cardiovascular magnetic resonance"[Title/Abstract]) OR CMR[Title/Abstract]) OR LGE[Title/Abstract]) OR "late gadolinium enhancement"[Title/Abstract]) OR gadolinium[Text Word]) OR "delayed enhancement"[Title/Abstract]) OR "T1 time"[Title/Abstract]) OR "T1 mapping"[Title/Abstract] | 254777 |
| #1 | Search (((heart diseases[MeSH Terms]) OR cardiomyopathy[MeSH Terms]) OR amyloid*[Title/Abstract]) OR CA[Title/Abstract] | 1162544 |

1. Biosis Preview (web of science)


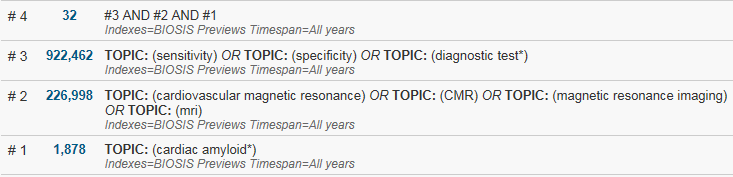


1. Embase


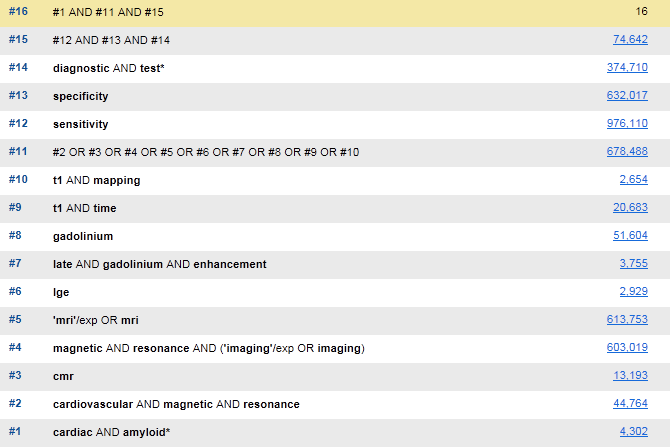


1. ISI web of science


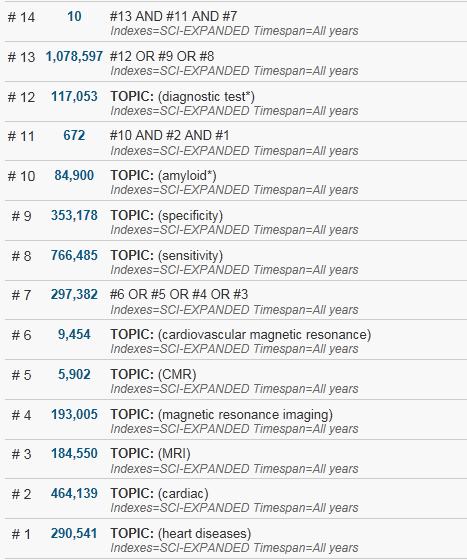


1. Cochrane library


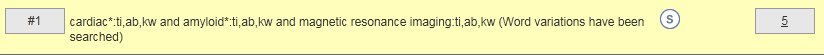


1. CNKI

#1 cardiac amyloidosis[Title]

#2 cardiac amyloidosis[Keywords]

#3 #1 OR #2

#4 magnetic resonance imaging[Title]

#5 magnetic resonance imaging[Keywords]

#6 #4 OR #5

#7 #3 AND #6
