# Supplementary material for: Diagnostic accuracy of cardiovascular magnetic resonance for patients with suspected cardiac amyloidosis: a systematic review and meta-analysis
Source: BMC Cardiovasc Disord. 2016 Jun 7;16:129. doi: 10.1186/s12872-016-0311-6 (PMC4897958; doi:10.1186/s12872-016-0311-6)
Supplement: Additional file 2: — Summary indexes in the meta-analysis. (DOCX 15 kb) [file 12872_2016_311_MOESM2_ESM.docx]

**Additional file 2**

Summary indexes in the meta-analysis

**Summary Positive Likelihood Ratio (Random effects model)**

Study | LR+ [95% Conf. Iterval.] % Weight

--------------------------------------------------------------------------------------------

Holger Vogelsberg | 14.400 2.108 - 98.367 16.18

James A. White | 3.045 1.276 - 7.264 32.66

Bethany A. Austin | 9.265 2.451 - 35.018 24.17

Frederick L. Ruberg | 6.000 0.969 - 37.141 17.27

Giovanni Donato Aqua | 44.906 2.870 - 702.59 9.71

--------------------------------------------------------------------------------------------

**(REM) pooled LR+ | 7.481 2.835 - 19.739**

--------------------------------------------------------------------------------------------

Heterogeneity chi-squared = 7.67 (d.f.= 4) p = 0.105

Inconsistency (I-square) = 47.8 %

Estimate of between-study variance (Tau-squared) = 0.5534

No. studies = 5.

Filter OFF

Add 1/2 to all cells of the studies with zero

**Summary Negative Likelihood Ratio (Random effects model)**

Study | LR- [95% Conf. Iterval.] % Weight

--------------------------------------------------------------------------------------------

Holger Vogelsberg | 0.212 0.076 - 0.586 16.34

James A. White | 0.046 0.003 - 0.723 2.23

Bethany A. Austin | 0.130 0.035 - 0.482 9.89

Frederick L. Ruberg | 0.167 0.056 - 0.496 14.25

Giovanni Donato Aqua | 0.202 0.117 - 0.348 57.28

--------------------------------------------------------------------------------------------

**(REM) pooled LR- | 0.183 0.121 - 0.277**

--------------------------------------------------------------------------------------------

Heterogeneity chi-squared = 1.60 (d.f.= 4) p = 0.809

Inconsistency (I-square) = 0.0 %

Estimate of between-study variance (Tau-squared) = 0.0000

No. studies = 5.

Filter OFF

Add 1/2 to all cells of the studies with zero

**Analysis of Diagnostic Threshold**

--------------------------------------------------------------------------------

Spearman correlation coefficient: 0.800 p-value= 0.104

(Logit(TPR) vs Logit(FPR)

--------------------------------------------------------------------------------

Moses' model (D = a + bS)

Weighted regression (Inverse Variance)

Var Coeff. Std. Error T p-value

--------------------------------------------------------------------------------

a 4.189 0.593 7.067 0.0058

b( 1) -0.201 0.380 0.530 0.6327

--------------------------------------------------------------------------------

Tau-squared estimate = 0.0000 (Convergence is achieved after 1 iterations)

Restricted Maximum Likelihood estimation (REML)

No. studies = 5

Filter OFF

Add 1/2 to all cells of the studies with zero

**Summary Sensitivity**

Study | Sen [95% Conf. Iterval.] TP/(TP+FN) TN/(TN+FP)

--------------------------------------------------------------------------------------------

Holger Vogelsberg | 0.800 0.519 - 0.957 12/15 17/18

James A. White | 1.000 0.782 - 1.000 15/15 7/10

Bethany A. Austin | 0.882 0.636 - 0.985 15/17 19/21

Frederick L. Ruberg | 0.857 0.637 - 0.970 18/21 6/7

--------------------------------------------------------------------------------------------

**Pooled Sen | 0.882 0.781 - 0.948**

--------------------------------------------------------------------------------------------

Heterogeneity chi-squared = 4.71 (d.f.= 3) p = 0.194

Inconsistency (I-square) = 36.3 %

No. studies = 4.

Filter OFF

Add 1/2 to all cells of the studies with zero

**Summary Specificity**

Study | Spe [95% Conf. Iterval.] TP/(TP+FN) TN/(TN+FP)

--------------------------------------------------------------------------------------------

Holger Vogelsberg | 0.944 0.727 - 0.999 12/15 17/18

James A. White | 0.700 0.348 - 0.933 15/15 7/10

Bethany A. Austin | 0.905 0.696 - 0.988 15/17 19/21

Frederick L. Ruberg | 0.857 0.421 - 0.996 18/21 6/7

--------------------------------------------------------------------------------------------

**Pooled Spe | 0.875 0.759 - 0.948**

--------------------------------------------------------------------------------------------

Heterogeneity chi-squared = 3.31 (d.f.= 3) p = 0.347

Inconsistency (I-square) = 9.3 %

No. studies = 4.

Filter OFF

Add 1/2 to all cells of the studies with zero
